# High prevalence of sexual *Chlamydia trachomatis* infection in young women from Marajó Island, in the Brazilian Amazon

**DOI:** 10.1371/journal.pone.0207853

**Published:** 2018-11-29

**Authors:** Leonardo Miranda dos Santos, Maria Renata Mendonça dos Santos Vieira, Jéssica Fernanda Galdino Oliveira, Josinaide Quaresma Trindade, Danielle Murici Brasiliense, Stephen Francis Ferrari, Mihoko Yamamoto Tsutsumi, Hellen Thais Fuzii, Edivaldo Costa Sousa Junior, Edna Aoba Yassui Ishikawa, Ricardo Ishak, Maísa Silva de Sousa

**Affiliations:** 1 Laboratório de Biologia Molecular e Celular, Núcleo de Medicina Tropical, Universidade Federal do Pará, Belém, Pará, Brasil; 2 Programa de Pós-graduação em Doenças Tropicais, Universidade Federal do Pará, Belém, Pará, Brasil; 3 Programa de Pós-graduação em Análises Clínicas, Universidade Federal do Pará, Belém, Pará, Brasil; 4 Laboratório de Citopatologia, Instituto de Ciências Biológicas, Universidade Federal do Pará, Belém, Pará, Brasi; 5 Seção de Bacteriologia e Micologia, Instituto Evandro Chagas, Ananindeua, Pará, Brasil; 6 Departamento de Ecologia, Universidade Federal do Sergipe, São Cristóvão, Brasil; 7 Laboratório de Imunopatologia, Núcleo de Medicina Tropical, Universidade Federal do Pará, Belém, Pará, Brasil; 8 Seção de Virologia, Instituto Evandro Chagas, Ananindeua, Pará, Brasil; 9 Laboratório de Virologia, Instituto de Ciências Biológicas, Universidade Federal do Pará, Belém, Pará, Brasil; University of Illinois at Urbana-Champaign, UNITED STATES

## Abstract

**Background:**

*Chlamydia trachomatis* is the most prevalent bacterial sexually transmitted infection (STI) in the world. Approximately 80% of infected women are asymptomatic, although this infection can lead to serious complications in the female reproductive tract. Few data on *Chlamydia* infection are available in rural Amazonian communities.

**Objectives:**

To evaluate the prevalence of sexual *C*. *trachomatis* infection in women from Marajó Archipelago communities in the Amazon region of Brazil and to identify associated factors and genotypes.

**Methods:**

We utilized amplification of the *ompA* gene by nested PCR. Positive samples were genotyped by sequencing. Study participants completed a questionnaire on social, epidemiological, and reproductive health variables. A Poisson regression was used to evaluate the degree of association of these variables with the infection.

**Results:**

The sexual infection by *C*. *trachomatis* was observed in 4% (16/393) of the subjects, and was more often found in women aged ≤25 (14.3%; 95% CI = 2.83–35.47; p <0.001), and in women with a household income of less than one Brazilian monthly minimum wage (5.2%; 95% CI = 1.33–11.37; p = 0.014). The *ompA* gene was sequenced in 13 samples, revealing F genotypes (38.4%, n = 5), D (23%, n = 3), E (15.3%, n = 2), Ia (7.6%, N = 1), J (7.6%, n = 1) and B (7.6%, n = 1).

**Conclusions:**

We recorded a high prevalence of sexual infection by *C*. *trachomatis* in young and poor women from the interior of the Brazilian Amazon. This high prevalence and the frequencies of the main genotypes were similar to those found in major Brazilian urban centers. Our results reinforce the importance of the screening of this neglected infection, and the prevention of later sequelae in young women from rural and urban areas of Brazil.

## Introduction

*Chlamydia trachomatis* is the most prevalent bacterial Sexually Transmitted Infection (STI) in the world [1], with about 128 million cases being diagnosed annually [2]. The infection is asymptomatic in approximately 80% of the infected women, going completely unnoticed by most women. The lack of an early diagnosis may make it impossible to treat the disorder adequately, with serious consequences for the female reproductive tract, such as salpingitis, Pelvic Inflammatory Disease (PID), ectopic pregnancy and tubal infertility, with an annual cost of up to four billion dollars [3–6]. This infection is the most common cause of preventable infertility in sexually active women and is a risk factor for other STIs [7–10].

The *ompA* gene of *C*. *trachomatis* has 19 genotypes, which are associated with a range of pathologies in humans [11]. The A, B, Ba and C genotypes are associated with trachoma [12], while the D, Da, E, F, G, Ga, H, I, Ia, J and K genotypes are associated with non-invasive urogenital infections [13], and the L1, L2, L2a and L3 genotypes cause lymphogranuloma venereum [14]. In Brazil, the actual prevalence of this infection and the genotypes present in different populations are still unclear, making it difficult to understand the dynamics of infection and preventing the creation of effective *C*. *trachomatis* screening programs by Brazilian public health agencies. Previous studies using nucleic acid amplification techniques have demonstrated that *C*. *trachomatis* infection rates range from 5% to 31% in Brazilian youths and adults, and that the most frequent genotypes in the country are the E, F and D types [15–19].

In the Brazilian Amazon region, many communities are located far from major urban centers, and have little access to public healthcare services or other resources [19, 20]. Understanding the diversity and distribution of the genotypes is essential for the monitoring of the infection, although few studies are available on the genotypes of sexual *C*. *trachomatis* infections in Brazil, and only one has focused on an urban Amazonian population [18]. The vast Marajó Archipelago is located at the mouth of the Amazon River, in the Brazilian state of Pará, and is inhabited by a largely rural population, living in small communities with few public healthcare services or even basic programs focusing on reproductive health [21, 22]. The present study aimed to determine the prevalence of *C*. *trachomatis* and its genotypes in the endocervical infections of women that have limited access to public health services, resident in four communities of the Marajó Archipelago, and to describe the potential. Study participants completed a questionnaire on social, epidemiological, and reproductive health variables associated with the prevalence of this disease.

## Methods

### Study population and data collection

This cross-sectional study was conducted between March 2013 and May 2015. The target population included autochthonous women (n = 393), from four small communities [São Sebastião da Boa Vista (n = 113), Anajás (n = 96), Portel (n = 60) and Chaves (n = 124)] of the Marajó Archipelago in Pará, in the Brazilian Amazon region. This archipelago is considered to be one of Pará’s main tourist destinations. Its principal islands are Marajó, Caviana, Mexiana and Gurupá, with 16 municipalities and a large network of rivers, creeks, lakes, and marshlands that criss-cross the islands and impede terrestrial access to most communities.

During the study period, the multidisciplinary team travelled from Belém (Pará state capital) to the communities for the collection of data. The sexually-active women in each community were informed of the periodicity of the health exams and asked to seek gynecological care during the home visits of the designated municipal health agents.

We investigated a non-probabilistic, intentional, and conventional sample, composed of women 18 to 79 years old, who had either never had a Pap smear or who had last had one more than one year previously. Exclusion criteria were pregnancy, menstruation, and not wishing to participate in the study or not signing the informed consent form. The participants were asked to answer a questionnaire, and were made aware of the importance of providing reliable answers, in order to minimize possible bias. The following variables were investigated: age, conjugal status, occupation/education, household income, age at fist sexual intercourse, lifetime number of sexual partners, number of sexual partners in the past year, use of condoms, use of contraceptives, previous pregnancies, natural childbirth, miscarriage, and whether the Pap test was taken.

Cervical secretions were collected during routine pelvic examinations using an endocervical brush, and the samples were stored in cryogenic tubes containing 1 ml Tris-EDTA buffer (TE) [10 mM Tris-HCl pH 8.5; 1 mM EDTA] at a temperature of -20°C for testing.

### Ethics statement

This study was part of the "Epidemiological Markers in the Healthcare of the Marajó Archipelago" project, which was approved by the Ethics Committee for Research of the State Foundation for Hemotherapy, with authorization number 0003.0.324.000–10. All participants signed up to be adults, with no participants below the age of 18. Free and informed consent for participation in the study was obtained in writing before the collection of samples and epidemiological data. All the data were analyzed with complete anonymity.

### Extraction of the DNA

The DNA was extracted using a *pureLink Genomic DNA Purification*kit (*Invitrogen*, Carlsbad, CA, USA) according to the manufacturer's instructions and stored at -20°C until analysis. A Polymerase Chain Reaction (PCR) of the human *β* globin gene was conducted prior to the detection of *C*. *trachomatis* to confirm the suitability of the samples [23].

### Detection of the *ompA* gene of *C*. *trachomatis*

Detection of *C*. *trachomatis* was performed using a nested PCR protocol modified by Jalal et al. 2007 [24], which amplified 394bp of the *ompA* gene of *C*. *trachomatis*. The first reaction used 6.0 μL of *GoTaqGreen Master Mix* (Promega, Madison, WI, USA), 0.5 μL (containing 20 pmol/μL of each primer) of the primers P1 (A) (5'GACTTTGTTTTCGACCGTGTT-3 ') and P2 (5'AGCRTATTGGAAAGAAGCBCCTAA-3 '), 2 μL genomic DNA, and 3 μL sterile water for a final volume of 12 μL. The second reaction used 0.5 μL of the solution of the first reaction, 6.0 μL *Go Taq Green Master Mix* (Promega, Madison, WI, USA), 4.5 μL of sterile water, 0.5 μL (20 pmol/μL) of the primers P3 (5'-AAACWGATGTGAATAAAGARTT-3') and P4 (5'-TCCCASARAGCTGCDCGAGC-3'). In the two steps of the nested PCR, a negative and a positive control was used to optimize the result. In the first and second stages of the nested PCR, the initial activation was at 95°C for 5 min and 1 min, respectively, followed by 35 cycles of denaturation at 94°C for 40 s, annealing at 54°C for 30 s, and elongation at 72°C for 90 s, with a final extension step at 72°C for 7 min. The amplified products were visualized after electrophoresis in 1% agarose gel with ethidium bromide (0.5mg/mL) staining.

### DNA sequencing

The Sanger method of nucleotide sequencing was used. An approximately 990bp fragment of the *ompA* gene was amplified by nested PCR using primers P1(B) (5′-ATGAAAAAACTCTTGAAATCGG-3′) and OMP2 (5′-ACTGTAACTGCGTATTTGTCTG-3′), and whenever re-amplification was necessary, the inner primers MOMP87 (5′-TGA ACC AAG CCT TAT GAT CGA CGG A-3′) and RVS1059 (5′-GCA ATA CCG CAA GAT TTT CTA GAT TTC ATC-3′) were used [25]. The first step of the nested PCR was run in a 0.5 μL volume containing 20 pmol/μL of each primer P1(B) and OMP2 and 5.0 μL of the DNA extracted from the endocervical secretion, 14 μL of sterile water, 1.0 μL of MgCl_2_, 1.0 μL deoxynucleoside triphosphate (10mM), 2.5 μL of 10x buffer, and 0.5 μL of *Hotstar Taq DNA Polymerase* 1.5U (Qiagen). Amplification was run in a final reaction volume of 25 μl [25]. In the two steps of nested PCR a negative and a positive control was used to optimize the result, but these controls were not used in the sequencing.

In the initial step of the nested PCR, amplification conditions were 95°C for 5 min, followed by 40 cycles of 94°C for 30 s, 55°C for 30 s, and 72°C for 90 s, and a final extension at 72°C for 7 min. In the nested PCR, the MOMP87-RVS 1059 primer pair was used with 1.5 μl of the product of the first stage of the nested PCR, which was added to a final volume of 25 μl. The conditions of the second step of the nested PCR were the same as those described above, except for the annealing temperature which was 60°C, and the addition of 17.5 μl of sterile water [25].

The amplified products were visualized by ethidium bromide (0.5 mg/mL) staining after electrophoresis in 1% agarose gel. The products obtained by the nested PCR were purified using a *BigDye Xterminator Purification* kit (Applied Biosystems, Foster City, CA, USA) to sequence both strands. A *BigDyeTerminator Cycle* kit (Foster City, CA, USA) was used for the sequencing reaction, according to the manufacturer's instructions. The reaction mixtures were sequenced in an *ABI 3130* (Applied Biosystems, Foster City, CA, USA).

### Phylogenetic analysis and Genotyping

The sequences were assembled using the CAP3 software, aligned in MAFFT v 7.221 and edited in Bioinformatic Geneious v 8.1.7. The consensus sequences were compared with known *C*. *trachomatis* lineages [26] using the BLAST search tool in the National Center for Biotechnological Information (www.ncbi.nlm.nih.gov). The residues that correspond to the flanking primers were excluded from the analyses.

The phylogenetic analysis was run in three stages. The first stage involved the use of IQ-TREE v 1.3.2 for the selection of the most adequate evolutionary model for the maximum likelihood analysis. The phylogenetic reconstruction was also run in IQ-TREE. The standard error was obtained using a bootstrap value of 0.03 for 2000 repetitions. In the third stage, FigTree v 1.4.2 was used to edit the phylogenetic tree produced by the analyses.

### Statistical analysis

The data were analyzed using the Statistical Package for Social Sciences (SPSS) version 21.0 (SPSS, Chicago, illinois, EUA). A Poisson regression was used to examine the unadjusted and adjusted associations between the Prevalence Ratio (PR) and the different variables. A 95% confidence interval (CI) was estimated and a critical p value of 0.05 was considered in all analyses.

## Results

The ages of the 393 women investigated in the present study ranged from 18 to 79 years. The median (interquartile range) age was 40.0 (29.0–52.0) years, and 82.2% (n = 323) of the participants were older than 25 years. Most of the women (n = 68.7%) were married, 75.9% (n = 259) were employed informally, and 55.7% (n = 219) had a family income of no more than the Brazilian minimum wage (U$250–300/month). Just over half 51.1% of the women (n = 201) were not using oral contraceptives, 90.6% (n = 356) were already mothers, and 90.4% (n = 355) of these mothers reported having had a vaginal delivery. Around one third, 32.6% (n = 128), reported having suffered at least one miscarriage, and 81.4% regularly have Pap tests.

The total prevalence of sexual infection by *C*. *trachomatis* was 4.1% (16/393). The infection was detected in 14.3% of the women that were 25 years old or younger, with an adjusted prevalence 10 times higher (95% CI = 2.83–35.47; *p* <0.001) than in women older than 25 years. The prevalence of infection was also 3.88 times higher in women from low-income households (below minimum wage) in comparison with those from higher income households (95% CI = 1.33–11.37; *p* = 0.014). No other variables were associated statistically with *C*. *trachomatis* infection (Table 1).

**Table 1:** Social, epidemiological, and reproductive health variables characteristics, with raw and adjusted prevalence ratios, in women from communities in Marajó Archipelago, Pará, Brazil.

Social Variables			Total (n = 393)		*C*. *trachomatis* positive 4.1% (16/393)		RP Ajusted (CI_95%_)	*p-value*
			n	%	n	%		
	Age (years)^a^	≤25	70	17.8	10	14.3	10.01(2.83–35.47)	<0.001*
		>25	323	82.2	6	1.8	1	
		Not answered	0	0				
	Conjugal status^a^	Single	96	24.4	7	7.3	1.72(0.54–5.53)	0.383
		Married	270	68.7	6	2.2	1	
		Not answered	27	6.9				
	Occupation^a^	Informal	259	65.9	8	3.1	0.59(0.21–1.65)	0.312
		Student	105	26.7	6	5.7	1	
		Not answered	29	7.4				
	Household income^a^ (number of Brazilian minimum wages)	<1	154	39.2	8	5.2	3.88(1.33–11.37)	0.014*
		≥1	219	55.7	8	3.7	1	
		Not answered	20	5.1				
Epidemiological variables	Age at fist sexual intercourse (years)^b^	<15	127	32.3	9	7.1	2.93(0.91–9.47)	0.073
		≥15	239	60.8	7	2.9	1	
		Not answered	27	6.9				
	Sexual partner^a^	Yes	285	72.5	9	3.1	2.78(0.74–10.49)	0.132
		No	94	23.9	7	7.4	1	
		Not answered	14	3.6				
	Sexual partners in the last year^b^	>1	100	25.4	2	2	1.03(0.13–7.95)	0.974
		1	238	60.6	10	4.2	1	
		Not answered	55	14.0				
	Condom use^a^ ^b^	Yes	59	15.0	4	6.8	1.33(0.28–6.25)	0.715
		No	321	81.7	12	3.7	1	
		Not answered	13	3.3				
	Number of sexual partners in life^b^	>3	75	19.1	6	8.0	1.94(0.51–7.44)	0.329
		≤3	239	60.8	8	3.3	1	
		Not answered	79	20.1				
Reproductive health variables	Contraceptive use^a^	Yes	188	47.9	7	3.7	0.92(0.31–2.76)	0.891
		No	201	51.1	9	4.4	1	
		Not answered	4	1				
	Previous pregnancy^b^	Yes	356	90.6	13	3.7	0.59(0.13–2.58)	0.484
		No	32	8.1	2	6.5	1	
		Not answered	5	1.3				
	Natural childbirth^b^	Yes	355	90.3	13	3.7	0.63(0.14–2.79)	0.543
		No	34	8.7	2	5.9	1	
		Not answered	4	1.0				
	Miscarriage ^b^	Yes	128	32.6	3	2.3	0.53(0.15–1.90)	0.328
		No	260	66.1	12	4.6	1	
		Not answered	5	1.3				
	Pap smear test^b^	Yes	320	81.4	12	3.8	0.85(0.21–3.38)	0.818
		No	69	17.6	3	4.3	1	
		Not answered	4	1.0				

RP Adjusted (ratio of adjusted prevalence): variables adjusted to each other in each group—multiple analysis. 95% CI: 95% Confidence Interval.

*: Statistically significant *p value*

^a^:Current variables

^b^:Anamnesis variables.

**Fig 1:**
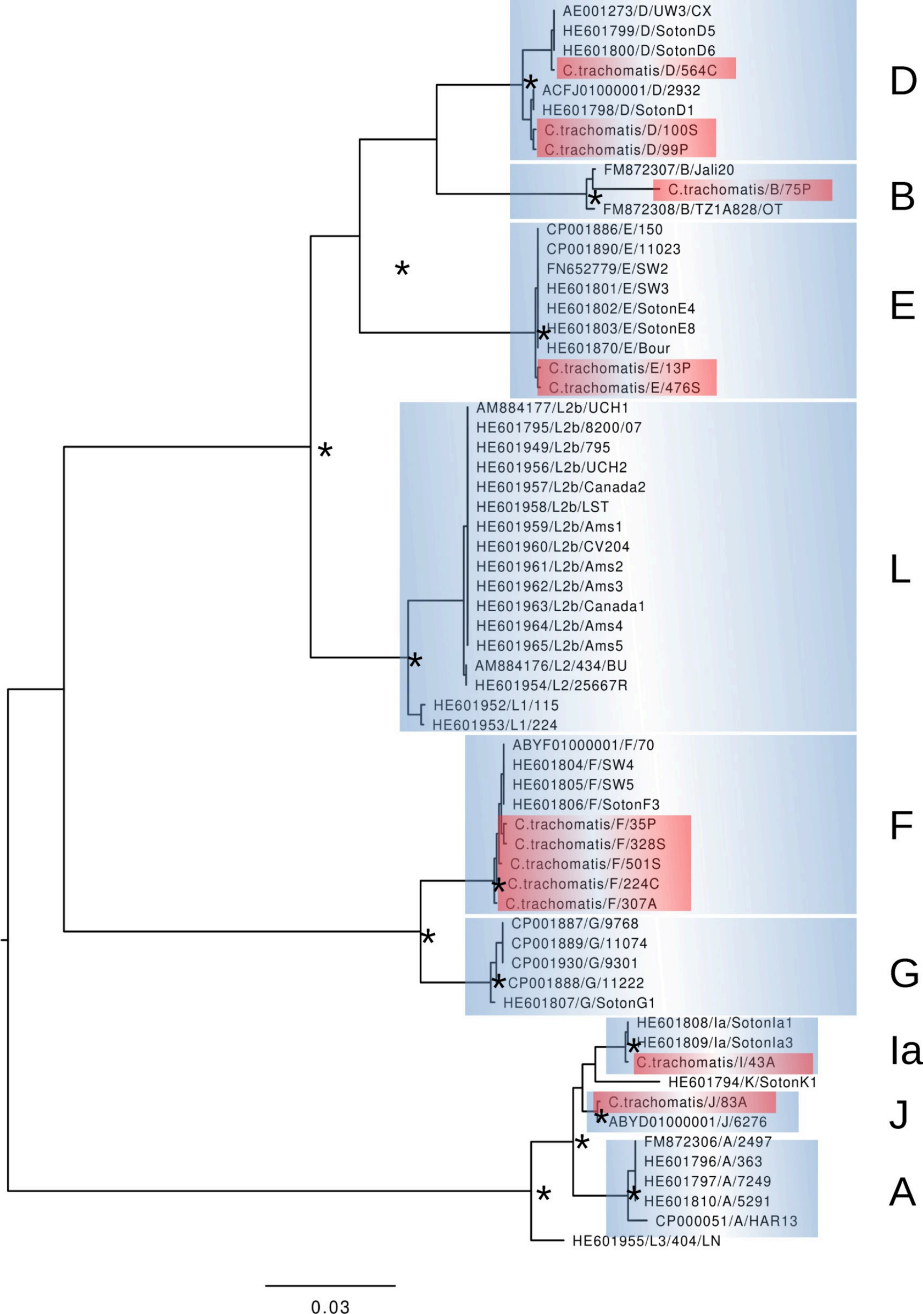
Results of the phylogenetic analysis of the *ompA* gene sequences of *C*. *trachomatis* detected in the endocervical samples of women from the Marajó Archipelago, Pará, Brazil. The samples analyzed in the present study are shown in red, and all other were obtained from GenBank (https://www.ncbi.nlm.nih.gov/genbank).

The *ompA* gene was sequenced in 13 of the 16 positive samples. Six genotypes were detected F (38.4%, n = 5), D (23%, n = 3), and E (15.3%, n = 2), with one sample each (7.6%) of the Ia, J, and B genotypes. The phylogenetic analysis revealed the evolutionary relationship among the *C*. *trachomatis* genotypes from the present study, with 99–100% similarity among the lineages, and 87.9% of similarity with the *C*. *trachomatis* reference lineage (Fig 1). The nucleotide sequences of the different *C*. *trachomatis* lineages identified in the present study were deposited in GenBank (NCBI) under access codes KU295204–KU295216. (Figs 1 and S1)

## Discussion

This preliminary study presented a molecular approach for the diagnosis of *C*. *trachomatis* infection in women who are socially deprived, residents in communities with little or no access to health care and laboratory procedures, which not only identified infection rates, but also the genotypes involved. The study participants completed a questionnaire on social, epidemiological, and reproductive health variables associated with the prevalence of this disease.

The low prevalence (4.1%) of *C*. *trachomatis* sexual infection identified in these communities may be related to the maturity of the women surveyed (mean age of 41 years and 82.2% more than 25 years old). On the other hand, we identified a significantly higher prevalence (14.3%) of *C*. *trachomatis* infection in young women (≤ 25 years old). High prevalence (11%) [18] and (18%) [19] were also identified in young women from the region’s largest city (Belém).

Access to health services is precarious in some parts of northern Brazil, such as Marajó Island, mainly due to its geographical characteristics [20,21]. In addition to being more common in younger women, the infection was also associated with a low family income.

In urban areas of other Brazil regions, infection rates vary from 9.6% to 31.0% [17, 27–32].

Even at such high prevalence rates, the association with risk variables is not easily identified, but there is evidence linking prevalence to young age and sexual conduct [29], as well as low income [30]. Precarious social conditions, a lack of economic opportunities, and risky sexual behavior are all closely associated with the incidence of STIs in young people [32].

High rates of sexual infection by *C*. *trachomatis* were recorded in Spain (8.5%) [33] and Great Britain (12.3%) [34]. Monitoring urogenital *C*. *trachomatis* infection by DNA amplification in women of less than 25 years of age should be a priority in most of these countries [35–38].

Although three samples could not be sequenced, six genotypes were identified in the remaining 13 samples. In general, our findings confirmed a higher prevalence of *C*. *trachomatis* genotypes F, D, E in sexual infections, as already described in an urban study in northern Brazil, together with genotype J [18]. Previous serological studies in the Amazon have shown that the distribution of *C*. *trachomatis* serotypes is similar to that observed in the present study [22]. Similar *C*. *trachomatis* genotypes have also been found in other regions of the Brazil [15–16] and the world [39–42].

The present study identified the occurrence of the B genotype of *C*. *trachomatis* in sexual infections of women from rural communities in the Brazilian Amazon region. The presence of the B genotype of *C*. *trachomatis*, an ocular genotype, is not unusual in the genital samples and has been reported in other studies [43–45]. Trachoma infection is another prominent clinical feature of *C*. *trachomatis* infection that has been reported from the Marajó Archipelago [46, 47]. However, it is still too early to confirm that this finding is related to cross-infection or gene recombination, which were not investigated specifically in the present study.

The lack of a public health program for the screening of this STI in Brazil is a major obstacle to the understanding of the epidemiology of *C*. *trachomatis* in this country. This favors the onset of reproductive problems, which can develop slowly and silently [48, 49].

The molecular screening for *C*. *trachomatis* infection is not yet included in the National Strategic Plan for STDs and AIDS in Brazil [50]. However, it would be important to include techniques with high sensitivity and specificity for the primary screening of active infection by *C*. *trachomatis*. More studies on the molecular prevalence, cross-infection, gene recombination, and genotypes of *C*. *trachomatis* will also be important, especially in young women, to elucidate the epidemiological network of this neglected STI.

The principal limitations of this study may be related to the relatively reduced sample size in comparison with the vast universe of communities in the Brazilian Amazon region, together with possible social biases in the responses of the women to the questionnaire. In the context of these considerations, this preliminary study is part of a larger project that aims to identify potential indicators that will support effective strategies for the improvement of the quality of life of the populations of the Marajó Archipelago. While it should be possible to extrapolate these findings to other, similar Amazonian communities, further research will be important to elucidate the exact nature of this infection and the distribution of the sexual and ocular genotypes of *C*. *trachomatis* in the region.

## Conclusions

We report a high prevalence of sexual infection by *C*. *trachomatis* in young and poor women from the interior of the Brazilian Amazon region. This prevalence and the diversity of *C*. *trachomatis* genotypes identified in this study were similar to that found in other Brazilian regions. Our results reinforce the importance of the adequate screening of this infection for the prevention of late sequelae in the young population of rural and urban areas of Brazil.

## Supporting information

S1 Fig Phylogenetic analysis of the 990 bps nucleotide sequence of the *ompA* gene of *C*. *trachomatis*. The 13 samples sequenced in this study are shown in the tree in red letters.
(DOCX)
