# Supplementary material for: High prevalence of sexual Chlamydia trachomatis infection in young women from Marajó Island, in the Brazilian Amazon
Source: PLoS One. 2018 Nov 29;13(11):e0207853. doi: 10.1371/journal.pone.0207853 (PMC6264820; doi:10.1371/journal.pone.0207853)
Supplement: S1 Fig — The 13 samples sequenced in this study are shown in the tree in red letters. (DOCX) [file pone.0207853.s001.docx]

| S1: Accession numbers of nucleotide sequences of *ompA* gene fragment of the *Chlamydia trachomatis*. These sequences are posted at www.ncbi.nlm.nih.gov/genbank | | | |
| --- | --- | --- | --- |
| Access Number | Strain | Genotype | County* |
| KU295204 | 75P | B | Portel |
| KU295205 | 99P | D | Portel |
| KU295206 | 100S | D | São Sebastião da Boa vista |
| KU295207 | 564C | D | Chaves |
| KU295208 | 13P | E | Portel |
| KU295209 | 476S | E | São Sebastião da Boa vista |
| KU295210 | 35P | F | Portel |
| KU295211 | 224C | F | Chaves |
| KU295212 | 307A | F | Anajás |
| KU295213 | 328S | F | São Sebastião da Boa vista |
| KU295214 | 501S | F | São Sebastião da Boa vista |
| KU295215 | 43A | I | Anajás |
| KU295216 | 83A | J | Anajás |
| *Marajó Island, Para State, Brazil. | | | |
